# Mucosal Leishmaniasis Caused by *Leishmania (Viannia) braziliensis* and *Leishmania (Viannia) guyanensis* in the Brazilian Amazon

**DOI:** 10.1371/journal.pntd.0000980

**Published:** 2011-03-08

**Authors:** Jorge Augusto de Oliveira Guerra, Suzane Ribeiro Prestes, Henrique Silveira, Leila Inês de Aguiar Raposo Câmara Coelho, Pricila Gama, Aristoteles Moura, Valdir Amato, Maria das Graças Vale Barbosa, Luiz Carlos de Lima Ferreira

**Affiliations:** 1 Tropical Medicine Foundation of Amazonas, Manaus, Amazonas, Brazil; 2 University of the State of Amazonas, Manaus, Amazonas, Brazil; 3 Center for Malaria and other Tropical Diseases, UEI Malaria, Institute of Hygiene and Tropical Medicine of Lisboa, New University of Lisboa, Lisboa, Portugal; 4 Federal University of Amazonas, Manaus, Amazonas, Brazil; 5 University of São Paulo, São Paulo, São Paulo, Brazil; Hospital Universitário, Brazil

## Abstract

**Background:**

*Leishmania (Viannia) braziliensis* is a parasite recognized as the most important etiologic agent of mucosal leishmaniasis (ML) in the New World. In Amazonia, seven different species of *Leishmania*, etiologic agents of human Cutaneous Leishmaniasis, have been described. Isolated cases of ML have been described for several different species of *Leishmania*: *L. (V.) panamensis*, *L. (V.) guyanensis* and *L. (L.) amazonensis*.

**Methodology:**

*Leishmania* species were characterized by polymerase chain reaction (PCR) of tissues taken from mucosal biopsies of Amazonian patients who were diagnosed with ML and treated at the Tropical Medicine Foundation of Amazonas (FMTAM) in Manaus, Amazonas state, Brazil. Samples were obtained retrospectively from the pathology laboratory and prospectively from patients attending the aforementioned tertiary care unit.

**Results:**

This study reports 46 cases of ML along with their geographical origin, 30 cases caused by *L. (V.) braziliensis* and 16 cases by *L. (V.) guyanensis*. This is the first record of ML cases in 16 different municipalities in the state of Amazonas and of simultaneous detection of both species in 4 municipalities of this state. It is also the first record of ML caused by *L. (V.) guyanensis* in the states of Pará, Acre, and Rondônia and cases of ML caused by *L. (V.) braziliensis* in the state of Rondônia.

**Conclusions/Significance:**

*L. (V.) braziliensis* is the predominant species that causes ML in the Amazon region. However, contrary to previous studies, *L. (V.) guyanensis* is also a significant causative agent of ML within the region. The clinical and epidemiological expression of ML in the Manaus region is similar to the rest of the country, although the majority of ML cases are found south of the Amazon River.

**Author Summary**

Leishmaniasis is considered a neglected disease with 1.5 million new cases of cutaneous leishmaniasis (CL) occurring each year. In the Amazon region and in the Americas in general, ML is caused by *Leishmania (Viannia) braziliensis*, though in rare cases it has been related to other species. ML, which is associated with inadequate treatment of CL, normally manifests itself years after the occurrence of CL. Clinical features evolve slowly and most often affect the nasal cavity, in some cases causing perforation, or even destruction, of the septum. Diagnosis is made using the Montenegro skin test, serology and histopathology of the patients' mucosal tissues, or by isolation of the parasites. PCR is the best way to identify the species of leishmaniasis and is therefore the diagnostic method of choice. This paper describes 46 cases of ML and their geographical origin, 30 cases associated with *L. (V.) braziliensis* and 16 with L. *(V.) guyanensis*. The species of leishmaniasis was identified using mucosal biopsies taken from Amazonian patients who were diagnosed and treated for ML in the tertiary care unit, in Manaus, Amazonas state, Brazil. This is the highest number of ML cases caused by *L. (V.) guyanensis* that has ever been reported.

## Introduction

Mucosal leishmaniasis (ML) in the Americas is mainly associated with *L. (V.) braziliensis*, the species recognized as the most important etiologic agent of the disease [1], [2]. Marzochi and Marzochi [3], based on the epidemiological and geographical distribution of that same species in different ecosystems, suggested that the human disease emerged in the Western Amazon, in particular south of the Amazon River, where *L. (V.) braziliensis* is the predominant form. Here the majority of patients with ML typically work in areas of primary rainforest, involved in activities related to forest product extraction [4], [5]; in these cases, the mucosal disease is the outcome of patients with a history of skin lesions that were not treated properly. Because of this, ML is an important public health problem and neglected disease in the Brazilian Amazon [5], [6]. *L. (V.) panamensis*, *L. (V.) guyanensis* and *L. (L.) amazonensis* have also been associated with ML, but very few cases of ML have been associated with *L. (V.) guyanensis* [7], [8], [9].

Early diagnosis and access to treatment of cutaneous leishmaniasis (CL) are crucial to avoid the development of ML and complications of this form of the disease, given its complexity and severity. In an attempt to improve diagnosis, molecular techniques such as Polymerase Chain Reaction (PCR) have been developed for the detection of *Leishmania* parasites in clinical samples [10], [11]; however, the low amount of DNA found in paraffin tissue hinders the characterization of species [12]. The identification of parasite species, today most commonly from genetic analyses, can directly contribute to our understanding of the epidemiology of leishmaniasis [13], [14], [15], [16]. The aim of this study is to describe the distribution of *Leishmania* species in Amazonian patients with ML that were treated at the Tropical Medicine Foundation of Amazonas (FMTAM), a tertiary care unit, while taking into consideration the geographical origin of each case.

## Materials and Methods

### Ethics Statement

This study was prepared in accordance with international ethical guidelines for biomedical research involving human subjects. The project was approved for retrospective and prospective study; retrospective study was from July 1992 to June 2006 and prospective study from July 2006 to December 2008. For the retrospective study, the samples (paraffin biopsies) were obtained from an already-existing collection in the pathology laboratory of FMTAM. For the prospective study, samples were obtained from patients presenting to FMTAM following informed consent, which was documented and signed.

### Study Design

The study population consists of patients with ML who were diagnosed and treated at the FMTAM in the city of Manaus, Amazonas state, Brazil, from July 1992 to December 2006. All patients came from the Brazilian Amazon. This region covers an area of 5,000,000 km2, 59% of Brazil's territory, and contains over 775 municipalities in the states of Amazonas, Amapá, Mato Grosso, Western Maranhão, Pará, Rondônia, Roraima, Acre and Tocantins. The total population for the region has been estimated at 20.3 million people – 68.9% of whom reside in urban areas while the remaining 31.1% reside in rural areas [17].

The distribution of cases was initially based on the municipality where patients with a prior history of CL acquired their cutaneous lesions that subsequently developed into mucosal disease. In patients with no prior history of CL the following exposure factors were considered to be more important than place of birth – living within an endemic area and a history of exposure factor activities in natural resource extraction in areas of natural forest.

### DNA Preparation

The biopsied tissues were preserved in three different media: a) formalin-fixed paraffin-embedded, b) imprint tissue on filter paper, or c) in buffer L6 [18]. The methodology for the extraction of DNA varied according to preservation methodology.

#### Embedded in paraffin

We performed 12 cuts of 20 µm in each block of embedded tissue using a disposable blade for each block. The samples were deposited in 1.5 mL Eppendorf tubes. The deparafinization was done with xilol and the DNA extraction using the protocol of the Dneasy Blood & Tissue Kit (Quiagen).

#### Filter paper

The material from the filter paper was cut and placed in sterile Eppendorf tubes. DNA was extracted using the “blood spot” protocol of the PureLink Genomic DNA kit (Invitrogen).

#### Biopsies solution L6

Excess solution was removed by centrifugation at 14.000 rpm. The supernatant was discarded and the tissue was homogenized using individual disposable test tubes (Anachem). The tissue was then processed using the DNeasy Blood & Tissue Kit (Qiagen) as described by the manufacturer.

### DNA Amplification by PCR

The presence of *Leishmania* DNA in tissue samples was detected by PCR using genus-specific primer 13a and 13b [19] according to the protocol described by Reale et al. [20].

In all tissue samples that were positive for *Leishmania*, PCR-RPLF was used to identify each species present in the biopsy. PCR was performed as described by Marfurt et al. [21]. DNA was amplified using primers Fme and Rme. Ten µl of the PCR products were digested with 1 U HaeIII and 1 U NcoI (New England Biolabs) at 37°C for 2 hours and 30 minutes. The resulting restriction fragments were separated on a 2.5% agarose gel. The size of the fragments was estimated by comparison with a 100 bp DNA ladder and compared with positive controls for *L. (V.) braziliensis* and *L. (V.) guyanensis*.

As positive controls for DNA extraction, all DNA samples that did not amplify *Leishmania*-PCR using 13a and 13b primers were subjected to PCR targeting 147 bp fragments of human actin gene. The sequence of primer used was Hu_actin1_fwd 5′-CTGTGGCATCCACGAAACTA-3′ and Hu_actin1_rev 5′-AGGGCAGTGATCTCCTTCTG-3′. The PCR reaction was performed in a volume of 25µl, 18.75 µl H_2_O, 2.50 µl 10x buffer containing each primer 0.3 µl, 3.5µ MgCl2, 0.2 mM dNTPs and 1 U Platinum Taq DNA Polymerase (Invitrogen) 2 µl DNA template. The PCR conditions were 5 minutes at 94°C followed by 40 cycles of 35 seconds at 94°C, 30 seconds at 58°C and 30 seconds at 70°C, and a final extension at 70°C, 7 minutes.

## Results

**Figure 1:**
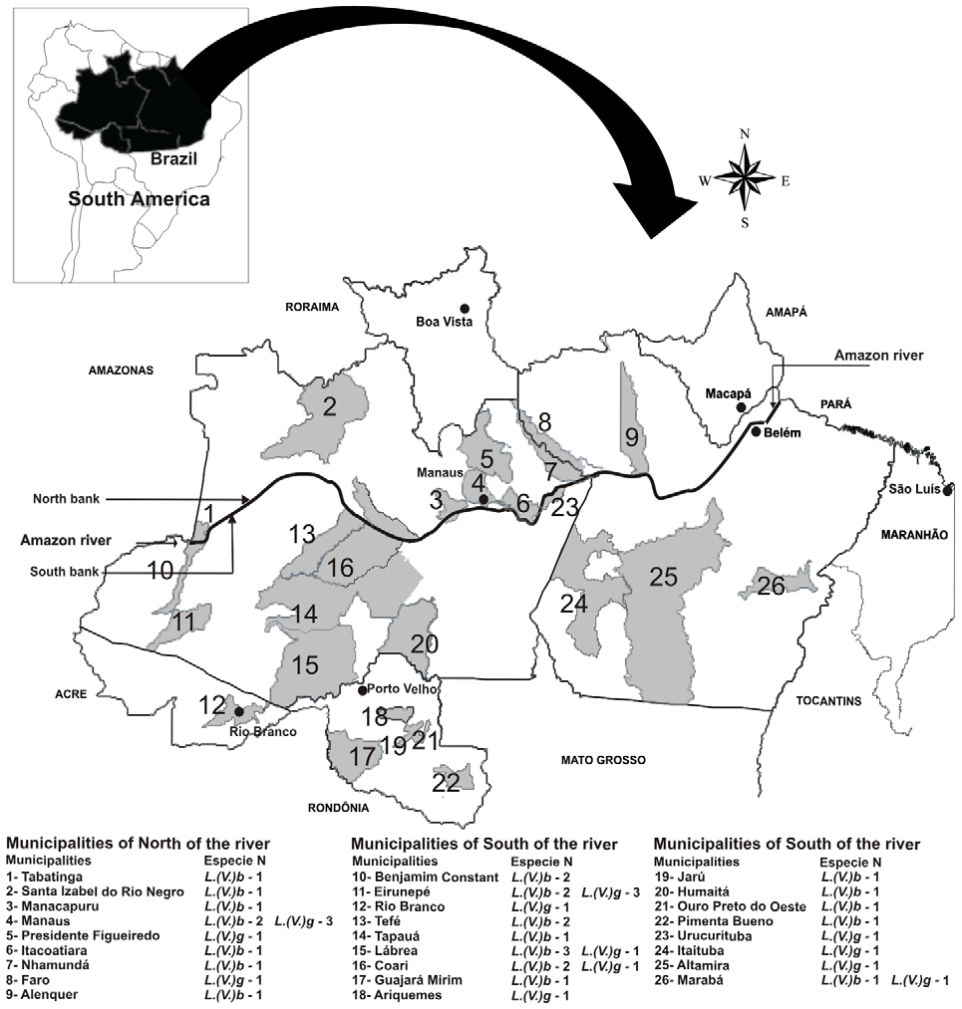
Spatial distribution of ML cases by species according to the municipality of origin.

The reported 46 cases of ML caused by *L. (V.) guyanensis* and *L. (V.) braziliensis*, along with their geographical origin, are depicted in Figure 1. This is the first record of ML cases in 16 different municipalities in the state of Amazonas and of simultaneous detection of both species in 4 municipalities of this state. It is also the first record of ML caused by *L. (V.) guyanensis* in the states of Pará, Acre, and Rondônia and cases of ML caused by *L. (V.) braziliensis* in the state of Rondônia. Thirty eight patients had a previous history of CL. Thirteen patients were from municipalities located north of the Amazon River and 33 patients came from south of the river.

Comparing the two characterized species revealed no differences concerning clinical and epidemiological aspects of cases studied (Table 1).

**Table 1:** Clinical and epidemiological aspects of cases according to the species identified.

Category	Subcategory	*L. (V.) braziliensis*	*L. (V.) guyanensis*
**History of CL**	Yes	24 (80%)	15 (93.8%)
	No	6 (20%)	1 (6.2%)
**Location of lesions**	Nasal	26 (86.7%)	11 (68.8%)
	Nasal/ oropharyx	3 (10%)	2 (12.5%)
	Nasal/ pharynx / larynx	1 (3.33%)	2 (12.5%)
	Oropharynx	0 (0%)	1 (6.2%)
**Clinical presentation**	Infiltrated	3 (10%)	3 (18.8%)
	Perforation	14 (46.7%)	6 (37.5%)
	Ulcer	13 (43.3%)	7 (43.8%)
	Positive	1 (3.33%)	2 (12.5%)
**Histop. examination**	Compatible with ML	23 (7.7%)	8 (62.5%)
	Inconclusive	1 (33.3%)	2 (12.5%)
	Not performed	2 (6.7%)	2 (12.5%)
	Non-specific chronic rhinitis	3 (10%)	2 (12.5%)
**Treatment of CL**	Regular	4 (16.7%)	5 (31.3%)
	Irregular	20 (83.3%)	10 (62.5%)
**Time between CL/ML**	Median time (years)	16.9	14.9
**Disease duration**	Median time (years)	12	8.6

*L. (V.) braziliensis – Leishmania (Viania) braziliensisL. (V.) guyanensis –Leishmania (Viannia) guyanensis*, CL-Cutaneous Leishmaniasis, Histop. Examination – Histopathological Examination, ML – Mucosal Leishmaniasis.

Within the group of patients studied, 39 had a history of previous CL and the source of origin of the mucosal disease was considered to be the same municipality where the cutaneous form was acquired. Of the seven patients without a previous history of CL, six lived and worked their whole lives in the same place and only went to Manaus for treatment; the remaining patient lived in a rural area of Manaus.

Among the 46 patients, 38 were male and 8 were female. The average period mediating skin to mucosal disease was 17.9 years (range: 4 months to 74 years) and the average duration of the mucosal disease was 8.3 years (30 days to 39 years). Two of the female patients had concomitant disease (CL/ML). Both were pregnant at the time of acquiring CL and therefore did not treat their skin lesions. The average age of the study population was 47.5 years (range: 16 to 80).

The PCR, performed on samples of 143 patients, was positive in 56 individuals, but in 10 samples it was not possible to characterize the species, probably due to the low amount of DNA or a consequence of formalin fixation and paraffin embedded tissues. Nine patients received adequate treatment of their CL while 29 had inadequate or irregular treatment. One patient had oral involvement alone and 45 had nasal involvement, eight of which were associated with oral forms. 20 patients had ulcerated or granulomatous lesions, 20 had perforated lesions, one of which involved the palate, and six patients had infiltration. The most frequent complaints were nasal obstruction (33/46), removal of crusts (28/46), epistaxis (18/46), rhinorrhea (16/46) and pruritus (16/46). The Montenegro skin test (MST) was positive in 41 cases, negative in one and not performed in the remaining four. Direct examination was positive in six cases, negative in 33 and not performed in six. The histopathological examination of the mucosal tissues was compatible with ML in 31 cases, positive in three and inconclusive in six. Chronic rhinitis was seen in three patients and absent in two. Serology was positive in 23 of 41 samples.

## Discussion

Leishmaniasis is a disease that is increasing in the Northern Hemisphere as a result of tourism and armed conflict in tropical regions [22], [23]. Cases of ML have been associated with multiple species [7], [8], [9], but our record of cases of ML caused by *L. (V.) guyanensis* is unusual. It was previously believed that the occurrence ML caused by *L. (V.) guyanensis* was extremely low, with only isolated cases having been described. The past low infection rate described in the literature is likely to be the result of limited studies on ML in the regions where *L. (V.) guaynensis* is endemic.

The geographic distribution of cases of American tegumentary leishmaniasis indicates that *L. (V.) braziliensis* is the predominant species south of the Amazon River [24], while studies in the Manaus region (north of the Amazon River) [18], [25] found that *L. (V.) guyanensis* is the most common species. In this study, 32 (71.1%) of the ML cases are from the south of the river: 22 (68.7%) were caused by *L. (V.) braziliensis* and 10 (31.3%) by *L. (V.) guyanensis*. North of the Amazon River, 13 (28.9%) patients were infected: 8 (61.5%) with *L. (V.) braziliensis* and 5 (38.5%) with *L. (V.) guyanensis* (Figure 1), which were mainly found in the Manaus area. This data suggests that no major differences exist between north and south of the river regarding the distribution of species causing ML.

The association between mucosal disease and previous skin lesions is widely accepted, as both forms can be caused by a single species [26], [27], and indeed in the 46 cases described here 37 had a previous history of CL. In the eastern Brazilian Amazon, mucosal disease occurs in patients with a history of previous skin lesions that were either untreated or treated inappropriately, and which were often caused by *L. (V.) braziliensis* [2], [26]. The data from this study on patient age, and the relationship between a previous history of CL and ML, are in support of previous findings [9], [28].

The current study extends this information and contributes new data on the distribution of *L. (V.) braziliensis* in western Amazonia, providing the first record of this species in 16 municipalities of Amazonas state and an additional 12 municipalities in three other states in the region: Acre – 1 case, Rondônia – 5 cases and Pará – 7 cases. It is also very important to emphasize the record of 16 ML cases caused by *L. (V.) guyanensis* in six different municipalities in Amazonas state, three in Pará, one in Rondônia and one in Acre (Figure 1).

It is probable that ML caused by *L. (V.) guyanensis* has always existed in Amazonia. We believe that this study fills a gap in knowledge about the epidemiology of ML, rather than identifying a change in disease pattern. Although this work has not assessed the genetic polymorphism of *L. (V.) guyanensis*, this has already been demonstrated [29], [30] and others have demonstrated this with respect to *L. (V.) braziliensis* [29], [31], [32], [33]. The finding of several hybrid genotypes of *Leishmania (Viannia)* in foci of cutaneous and mucocutaneous leishmaniasis has also been reported [34]. One cannot exclude the possibility of a genetic polymorphism of *L. (V.) guyanensis* in the etiology of ML in the Amazonian region, since little has been reported prior to this study.

The association between inappropriately treated cutaneous forms of the disease and the occurrence of ML appears to be maintained for both *L. (V.) guyanensis* and *L. (V.) braziliensis*. The association between cutaneous forms treated inappropriately and the occurrence of the mucosal form, also in ML caused by *L. (V.) guyanensis*, seems to keep the same relationship observed for the *L. (V.) braziliensis*. However, it should be noted that poor access to the diagnosis and treatment of leishmaniasis is common in the Amazon region. This is due to the isolation of communities, with access being almost exclusively by boat in many areas. Furthermore, many patients lack the financial resources to stay for long periods in Manaus to ensure adequate treatment and follow-up. These factors may be associated with the development of mucosal disease. The high prevalence in males in our study population has also been observed by other authors [35], [36]. The average time of 17.6 years between the diagnosis of CL and the appearance of ML (with one patient having a 74-yer gap between CL and ML) recorded in this study is also in agreement with previous findings [37], [38] on the persistence of this parasite in the host's body and the subsequent triggering of mucosal disease.

From a clinical point of view, we would like to draw the reader's attention to the large number of cases – 21 (45.7%) – with nasal perforation, which supports previous findings [27] that have demonstrated the potential of this species to cause more severe disease.

In summary, based on the results of this study, *L. (V.) braziliensis*, which caused 2/3 of the studied cases, is the predominant species that causes ML in the Amazon region. However, contrary to previous studies, *L. (V.) guyanensis* is also a significant causative agent of ML in the region. The clinical and epidemiological expression of ML in the Amazon region is similar to the rest of the country, although the majority of ML cases are found south of the Amazon River. ML infections are much more common in men than in women, and men also tend to develop more severe forms of disease with a high incidence of perforation and involvement of structures outside of the nasal cavity.

## Supporting Information

Checklist S1 Strobe Checklist
(0.06 MB DOC)
