# Supplementary material for: Mucosal Leishmaniasis Caused by Leishmania (Viannia) braziliensis and Leishmania (Viannia) guyanensis in the Brazilian Amazon
Source: PLoS Negl Trop Dis. 2011 Mar 8;5(3):e980. doi: 10.1371/journal.pntd.0000980 (PMC3050903; doi:10.1371/journal.pntd.0000980)
Supplement: Checklist S1 — Strobe Checklist (0.06 MB DOC) [file pntd.0000980.s001.doc]

**STROBE resposta**

**Title and abstract**

1 (a) commonly used term in the title or the abstract.

Mucosal, leishmaniasis, Leishmania (Viannia) braziliensis Leishmania (Viannia) Brazilian Amazon

(*b*) Provide in the abstract an informative and balanced summary of what was done

and what was found

This paper describes 46 cases of ML and their geographical origin, 30 for *Leishmania (Viannia) braziliensis* and 16 for *Leishmania (Viannia) guyanensis*, that were characterized by PCR of tissues taken from mucosal biopsies of Amazonian patients who were diagnosed with ML and treated in a Tertiary Care Unit, in the city of Manaus, Amazonas State, Brazil

and their geographical origin. This is the first record of ML cases in 16 different municipalities in the state of the Amazonas and of simultaneous detection of both species in 4 municipalities of this state. It is also the first record of LM by *L. (V.) guyanensis* in the states of Pará, Acre, and Rondônia and cases of LM by *L. (V.) braziliensis* in the state of Rondônia.

*L. (V.) braziliensis*, is the predominant species that causes ML in the Amazon region. However, contrary to the previous studies, *L. (V.) guyanensis* is also a significant causative agent of ML in the Amazonian Region.

**Introduction**

Background/rationale

2 Explain the scientific background and rationale for the investigation being reported

Leishmaniasis is considered a neglected disease with 1.5 million of Cutaneous Leishmaniasis (CL) cases a year. In the Amazon Region, and in the Americas in general, Mucocutaneous Leishmaniasis (ML) is caused by *Leishmania (Viannia) brazilensis*, though in rare cases it has been related to other species. This paper describes 46 cases of ML and their geographical origin, 30 cases associated with *L. (V.) braziliensis* and 16 with *L. (V.) guyanensis*. The species of leishmaniasis was identified using mucosal biopsies taken from Amazonian patients who were diagnosed and treated for ML in the Tertiary Care Unit, Manaus, Amazonas State, Brazil. This is the highest number of ML cases caused by *L. (V.) guyanensis* that has ever been reported. To date little is known about the distribution of species of *Leishmania* involved in cases of mucosal leishmaniasis in Amazonia. In literature there are only isolated case reports.

Objectives

3 State specific objectives, including any prespecified hypotheses

Described in accordance with the theoretical background.

1. Identify by PCR species of *Leishmania* agents of mucosal leishmaniasis in the Brazilian Amazon.
2. Evaluate the distribution of cases of mucosal leishmaniasis in the Amazon region in accordance with the species identified.
3. Clinically characterize cases identified according to species.
    
   Hypothesis: *Leishmania (V.) guyanensis* is an important agent of mucosal leishmaniasis in the Brazilian Amazon.

**Methods**

Study design

4 Present key elements of study design early in the paper

Selection of cases of leishmaniasis mucosa from the Brazilian Amazon.
Collection of material from mucosal biopsies.
Characterization of species per PCR.
Distribution of species according to the municipality of origin of cases.

Setting

5 Describe the setting, locations, and relevant dates, including periods of recruitment,

exposure, follow-up, and data collection

Dicionário - [Ver dicionário detalhado](http://www.google.com.br/dictionary?source=translation&hl=pt-BR&q=Explain how the study size was arrived at&langpair=en|pt)

This was a descriptive cross-sectional study whose population consists of patients with ML who were diagnosed and treated at the FMT-AM in the city of Manaus, Amazonas State, Brazil, from July 1992 to December 2006. All patients came from the Brazilian Amazon. This region covers an area of 5,000,000 km2, 59% of Brazil’s territory, and contains over 775 municipalities in the states of Amazonas, Amapá, Mato Grosso, Western Maranhão, Pará, Rondônia, Roraima, Acre and Tocantins.

Participants

6 (*a*) Give the eligibility criteria, and the sources and methods of selection of

Participants

The distribution of cases was initially based on the municipality where patients with a prior history of CL acquired their cutaneous lesions that subsequently developed into mucosal disease. In patients with no prior history of CL the following exposure factors were considered to be more important than place of birth – living within an endemic area and a history of exposure factor activities in natural resource extraction in areas of natural forest.

Variables

7 Clearly define all outcomes, exposures, predictors, potential confounders, and effect

modifiers. Give diagnostic criteria, if applicable

It was identified by PCR, 46 cases of mucosal leishmaniasis in four different Brazilian states, 16 *Leishmania (Viannia) guyanensis* and 30 *Leishmania (Vianna) braziliensis* as agents of disease in the Brazilian Amazon. The confounding factors could be associated with the origin of the cases because it is the patient's verbal information about the probable site of origin of the case. The identification technique was essential for the diagnosis of the species - was used PCR-RPLF to identify each species present in the biopsy. PCR was performed as described by Marfurt et al., 2003.

Data sources/

measurement

8* For each variable of interest, give sources of data and details of methods of assessment (measurement). Describe comparability of assessment methods if there is more than one group

Data on clinical and laboratory aspects of the cases were collected from medical records of patient care.

Bias

9 Describe any efforts to address potential sources of bias

The bias could be associated with the origin of the cases because it is the patient's verbal information about the probable site of origin of the case which is associated with species characterized.

Study size

10 Explain how the study size was arrived at

The study was descriptive, based on the findings of the species characterized in each patient, making the later correlation with the location of the origin of each case, not being applicable to the issue of sample size.

Quantitative variables

11 Explain how quantitative variables were handled in the analyses. If applicable,

describe which groupings were chosen and why

No statistical methods were applied in this study, except mean and median.

(*a*) Describe all statistical methods, including those used to control for confounding

(*b*) Describe any methods used to examine subgroups and interactions

(*c*) Explain how missing data were addressed

(*d*) If applicable, describe analytical methods taking account of sampling strategy

Statistical methods

12

(*e*) Describe any sensitivity analyses

Due to the similarity of results when comparing the percentages between groups of patients for *Leishmania (Viannia) guyanensis* and *Leishmania (Vianna) braziliensis* it was decided to do statistical analysis, because the percentages were clear for each species showing that there would be Statistical differences between the two groups.

Ouvir

Ler foneticamente

Dicionário - [Ver dicionário detalhado](http://www.google.com.br/dictionary?source=translation&hl=pt-BR&q=Explain how the study size was arrived at&langpair=en|pt)

**Results**

(a) Report numbers of individuals at each stage of study—eg numbers potentially

eligible, examined for eligibility, confirmed eligible, included in the study,

completing follow-up, and analysed

We included individuals from whom it had a biopsy of mucosal lesions that enabled the performance of PCR and included only those in the final evaluation of which was obtained a characterization of the species.
(B) Give Reasons for non-participation at each stage
We excluded patients for whom there was no possibility of doing PCR.

Participants

13*

(c) Consider use of a flow diagram

It was not necessary.

(a) Give characteristics of study participants (eg demographic, clinical, social) and

information on exposures and potential confounders

Demographic

- Age The average age of the study population was 47.5 years (range: from 16 to 80years)
- Sex - 38 were male and 8 were female.

Clinical

- Location of lesions - One patient had only oral involvement, 45 had nasal involvement, eight of which were associated with oral forms
- Clinical presentation - 20 patients had ulcerated or granulomatous lesions, 20 had perforation, one of which was in the palate, and six patients had infiltrated.
- Histopathological Examination - The histopathological exam of the mucosal tissues was compatible with LM in 31 cases, positive in three, and inconclusive in six.
- Treatment of CL - Nine patients received adequate treatment of their CL while 29 had inadequate or irregular treatment.
- Time between CL/ML - The average period mediating skin to mucosal disease was 17.9 years (range: 4 months to 74 years)
- Disease duration Median time (years) - The average duration of the mucosal disease was 8.3 years (30 days to 39 years).

Social

- Activity during the time of skin lesion or the time of exposure to the disease mucosa - The majority of patients with ML typically work in areas of primary rain forest, involved in activities related to forest product extraction
- History of CL - 39 patients had a history of previous Cutaneous Leishmaniasis.

Descriptive data

14*

(b) Indicate number of participants with missing data for each variable of interest

All patients had data on the variables of interest

Outcome data

15* Report numbers of outcome events or summary measures

(*a*) Give unadjusted estimates and, if applicable, confounder-adjusted estimates and

their precision (eg, 95% confidence interval). Make clear which confounders were

adjusted for and why they were included

(*b*) Report category boundaries when continuous variables were categorized

Not applicable

Main results

16

(*c*) If relevant, consider translating estimates of relative risk into absolute risk for a

meaningful time period

Not applicable

Other analyses

17 Report other analyses done—eg analyses of subgroups and interactions, and

sensitivity analyses

Not applicable

**Discussion**

Key results

18 Summarise key results with reference to study objectives

The results were in line with the objectives of the study was to describe the species involved in the Occurrence of mucosal leishmaniasis in patients from the Amazon.

Goal 1 - to characterize the species of Leishmania etiologic agents of Leishmaniose mucosa circulating in the Brazilian Amazon
Result: there were two species
Goal 2 - Show the distribution of cases according to their provenance
We identified 46 cases in four different states.
Goal 3 - Show the importance of *Leishmania (Viannia) guyanensis* and *Leishmania (Vianna) braziliensis* as agents of mucosal leishmaniasis in the Brazilian Amazon.
We characterized 30 cases of *Leishmania (Viannia) guyanensis* and 16 *Leishmania (Vianna) braziliensis* this Region.

Limitations

The limitation is the difficulty of obtaining positive for *Leishmania* at the species level from mucosal biopsies of patients diagnosed as LM, Leishmania in the presence of mucosal lesions is rare which decreases the sensitivity of the method, although it is now appointed as choice in the characterization of species of *Leishmania*.

Ouvir

Ler foneticamente

Dicionário - [Ver dicionário detalhado](http://www.google.com.br/dictionary?source=translation&hl=pt-BR&q=Explain how the study size was arrived at&langpair=en|pt)

19 Discuss limitations of the study, taking into account sources of potential bias or

imprecision. Discuss both direction and magnitude of any potential bias

The limitation in detecting the species using PCR, using mainly paraffin biopsies, may not allow the knowledge of the real range of species that cause disease in the Brazilian Amazon.

Interpretation

20 Give a cautious overall interpretation of results considering objectives, limitations,

multiplicity of analyses, results from similar studies, and other relevant evidence

Further studies are needed from cases of mucosal leishmaniasis from the region to define the species Associated with circulating disease. The study is tracking the new publication is planned for two years after this study.

Generalisability

21 Discuss the generalisability (external validity) of the study results

These results, although limited by difficulties in characterizing the samples can be validated by its unique character, because to date it was believed that the occurrence of *Leishmania (Viannia) guyanensis* was extremely rare in the Americas, this is the largest series of cases of leishmaniasis mucosa, etiologic diagnosis resulted from this region and the largest ever shown worldwide cases of mucosal leishmaniasis associated with *Leishmania (Viannia) guyanensis*.

Ouvir

Ler foneticamente

Dicionário - [Ver dicionário detalhado](http://www.google.com.br/dictionary?source=translation&hl=pt-BR&q=Explain how the study size was arrived at&langpair=en|pt)

**Other information**

Funding 22 Give the source of funding and the role of the funders for the present study and, if

applicable, for the original study on which the present article is based

*Give information separately for exposed and unexposed groups.
